# Increasing exercise adherence among elderly patients with chronic disease in primary care: a prospective cohort study

**DOI:** 10.1186/s12877-021-02572-5

**Published:** 2021-11-01

**Authors:** Seth VanDerVeer, Ronald Markert, Brant Bickford, Joseph Yuhas, Paul Pikman, Tim Wall, Kathryn Burtson

**Affiliations:** 1 Wright Patterson Medical Center, USAF, Wright State University Boonshoft School of Medicine, Dayton, USA; 2 Wright State University Boonshoft School of Medicine, Dayton, USA

## Abstract

**Background:**

Currently, the evidence for exercise in maintaining health, well-being, and physical functioning is overwhelming. Despite these benefits, more than 50% of the population fails to meet the recommended exercise requirements for age and health status. In our study, we sought to provide a method to increase exercise adherence that was both effective and time-efficient for physicians and their patients.

**Methods:**

The primary objective of this research study was to evaluate the effectiveness of a graded exercise protocol and biweekly monitoring on increasing the duration of aerobic exercise to 150 min per week in a population of elderly individuals with chronic disease. Secondarily, we evaluated for improvement in resting heart rate, blood pressure, body mass index (BMI), and cardiorespiratory fitness. The overall study design was a randomized, prospective cohort study with assessor blinding. Forty-five patients aged ≥60 years with multiple comorbidities were recruited from the Internal Medicine Clinic at Wright-Patterson AFB. Participants were randomized into a treatment or control arm and observed over a period of 34 weeks. Those in the treatment arm were given a graded walking protocol and received biweekly phone calls to evaluate compliance. Those in the control arm did not receive an intervention or biweekly monitoring. Measurements of heart rate, blood pressure, and BMI were taken quarterly in both groups. At the beginning and conclusion of the study, each participant completed a modified Balke treadmill test and Physical Activity Scale for the Elderly (PASE). Continuous variables were evaluated with the independent samples t-test, whereas categorical variables were evaluated with the chi-squared test.

**Results:**

A greater percentage of the treatment group achieved the primary outcome (41.6% vs. 0%; *p =* 0.003). Those in the treatment group also had favorable improvements in heart rate response (− 2.4 beats/min vs. + 5.3 beats/min; *p* = 0.038) and PASE (+ 66 vs.-20; *p* < 0.001). No significant differences were observed between groups for mean change in heart rate, blood pressure, or BMI.

**Conclusion:**

Guided, independent exercise and surveillance can be an effective tool in primary care practice to help patients reach the recommended levels of exercise for both age and health status.

## Introduction

There is an abundance of evidence supporting physical activity and physical fitness as effective in both lengthening and enhancing quality of life. Research has demonstrated that all body tissues and organ systems benefit from physical activity [1]. Despite these benefits, only a fraction of the population meets minimum exercise recommendations, and the percentage decreases further as individuals age. In 2015, the Center for Disease Control and Prevention reported that less than 45% of elderly individuals aged ≥65 years met the minimum aerobic exercise recommendations for age and health status; for those aged ≥85 years, the minimum recommendation was achieved by less than 20%. Within these groups, the lowest rates were seen among those with multiple comorbid conditions [2].

Multiple studies have shown that physicians are the preferred source of encouragement for exercise, and thus physicians are important in addressing the paucity of exercise compliance described above [3]. Exercise counseling by primary care physicians increases both interest and participation in exercise [4, 5]. Despite this opportunity, many physicians do not counsel or monitor the physical activity behaviors of their patients [6]. Perceived barriers to exercise counseling and monitoring include lack of time, knowledge, training, and instruments or materials. Consequently, we sought to provide a solution that was convenient and understandable for patients as well as effective and time-efficient for physicians.

The primary objective of this study was to evaluate the effectiveness of a graded exercise protocol with biweekly (every 2 weeks) monitoring on increasing the duration of aerobic exercise to 150 min per week in a population of elderly individuals with chronic disease. Secondarily, we evaluated improvement in several health predictors including heart rate, blood pressure, body mass index (BMI), and cardiorespiratory fitness. It was hypothesized that the use of a graded exercise protocol with biweekly monitoring would be superior to usual care in increasing compliance with the minimum American College of Sports Medicine (ACSM) aerobic exercise recommendations for age and health status.

## Methods

We performed a randomized cohort study with assessor blinding to evaluate the effect of a graded exercise protocol and biweekly monitoring on patient adherence to established exercise recommendations among elderly patients with chronic disease. The study was approved by the Wright Patterson AFB Medical Center (WPMC) Institutional Review Board (IRB). All participants provided written informed consent and HIPAA authorization. After a participant signed a HIPAA authorization, demographic and clinic data were retrieved from the outpatient clinic database.

Participants were recruited from Internal Medicine Clinic A at WPMC through print advertising and word-of-mouth. Inclusion criteria were: age ≥ 60 years, ability to ambulate independently without the assistance of a walker, and ability to perform ambulation for ≥10 min at a slow pace on a level surface. All potentially eligible participants were invited to undergo an assessment of cardiorespiratory fitness with a modified Balke treadmill test. The modified Balke treadmill test was chosen for its safety and efficacy as a submaximal test of aerobic capacity in the elderly population [7]. The treadmill was set at 2.0 mph with the grade starting at 0% and increasing by 2% every 3 min to a maximum of 10% over a total of 6 stages. Heart rate was recorded at 3-min intervals, which coincided with the conclusion of each stage. Subjects were excluded from the study if they failed to safely ambulate at 2.0 mph during the modified Balke test.

A blinded statistician subsequently performed a computer-generated randomization sequence for subject allocation. Participants were randomly allocated to the treatment or control group and then scheduled for an appointment at Internal Medicine Clinic A. Baseline measurements of heart rate, blood pressure, and BMI were taken. Participants completed a Physical Activity Scale for the Elderly (PASE), which provided a qualitative assessment of baseline activity level. PASE is a comprehensive assessment of physical activity in the elderly, involving multiple domains ranging from recreational and work-related activities to household activities and caring for dependents. PASE has been validated in multiple studies over the past 20 years [8, 9].

Participants assigned to the treatment group received information and advice to complete an unsupervised, graded aerobic exercise protocol over a period of 34 weeks. The program featured walking at a low-intensity with the option of doing so on a treadmill at the gymnasium, at home, outside, or at another location such as a mall. Low-intensity activity was maintained through a self-administered talk test, which dictated that each participant was able to talk at all times during the activity. For each 2-week interval throughout the study, participants were given a designated walking time to complete. The program began with 2 days per week at 10 min per day and then progressed gradually. The number of exercise days was increased by 1 day every 8 weeks to a maximum of 5 days per week. The duration of daily exercise was increased by 1 min every 2 weeks until 22 min per day was achieved, and then by 2 min every 2 weeks to a maximum of 30 min per day.

No specific walking speed was prescribed since participants exhibited a wide range of baseline exertional capacity and potential for adaptive change. Table 1 shows that a greater increase in exercise activity was prescribed for the last 5 weeks since the participants were projected to have improved exercise capacity at this point. Table 1 outlines the graded aerobic exercise protocol. Participants in the treatment group were provided with a tracking sheet for their own accounting. They also received a short phone call every 2 weeks from a researcher to evaluate compliance. During each call, encouragement and confirmation of the designated walking time for the next 2-week interval were also provided.

**Table 1:** Graded aerobic exercise protocol

**Week 1–2**	**2 DAYS, 10 min**
**Week 3–4**	**2 DAYS, 11 min**
**Week 5–6**	**2 DAYS, 12 min**
**Week 7–8**	**2 DAYS, 13 min**
**Week 9–10**	**3 DAYS, 14 min**
**Week 11–12**	**3 DAYS, 15 min**
**Week 13–14**	**3 DAYS, 16 min**
**Week 15–16**	**3 DAYS, 17 min**
**Week 17–18**	**4 DAYS, 18 min**
**Week 19–20**	**4 DAYS, 19 min**
**Week 21–22**	**4 DAYS, 20 min**
**Week 23–24**	**4 DAYS, 21 min**
**Week 25–26**	**5 DAYS, 22 min**
**Week 27–28**	**5 DAYS, 24 min**
**Week 29–30**	**5 DAYS, 26 min**
**Week 31–32**	**5 DAYS, 28 min**
**Week 33–34**	**5 DAYS, 30 min**

Initial – 2 days per week; 10 min per day

Days – Increase by 1 day every 8 weeks to a maximum of 5 days/week

Minutes – Increase by 1 min every 2 weeks to reach 30 min/day

Walking speed – Variable to maintain low intensity activity for each individual

Participants assigned to the control group did not receive the graded aerobic exercise protocol or biweekly phone calls from staff. Instead, participants were instructed to follow the advice of their primary care physician regarding aerobic exercise. Since most primary care physicians do not place sufficient emphasis on the importance of physical activity, they typically do not counsel patients beyond repeating the current exercise recommendations. In turn, patients are usually left without a clear plan to reach the recommended level of physical activity. Therefore, the exercise advice of the participant’s own primary physician was selected to represent the standard of care for the control group.

In addition to the baseline assessments, both groups underwent quarterly vitals checks at week 12 and week 24 with repeat measurements of heart rate, blood pressure, and BMI. At the conclusion of the study, all patients underwent final testing, which included a vitals check, modified Balke treadmill test, and PASE. All testing followed a standard protocol.

The primary outcome was achievement of 150 min of aerobic exercise per week at the conclusion of the study. For those in the treatment group, exercise compliance was tracked biweekly as exercise duration was increased. Exercise compliance was rated on a scale of 0 to 100% at 25% intervals. Achievement of 150 min of weekly aerobic exercise during the last 2 weeks of the graded aerobic exercise protocol (week 33 or week 34) was credited as 100% compliance. Those in the control group who did not or were unable to attend final testing were contacted by telephone and asked if they were walking at least 150 min per week.

Secondary outcomes included changes in PASE and heart rate response on the modified Balke treadmill test. Both were measured during the baseline and final testing periods to evaluate for changes in overall activity level and improvements in cardiorespiratory fitness. To evaluate for improvement in heart rate response on the modified Balke test, the heart rate change between Stage 1 and Stage 6 from the baseline test was compared to that of the final test. In addition, changes in heart rate, blood pressure, and BMI at vitals check were prespecified endpoints. Measurements of these values were completed at baseline, two quarterly checks, and final testing.

Due to the frequency and nature of data collection, all participants were aware of their placement in either the treatment or control group. Similarly, researchers involved in data tracking on compliance for the treatment group were aware of a participant’s group placement. Additionally, the principal investigator, who participated in the measurement of vitals, modified Balke testing, and PASE, was aware of the group designation for each participant. However, WPMC technicians who participated in the baseline and first quarter vitals check were not aware of a participant’s group designation. Further, the statistician, who performed all statistical analyses, was blinded to participant identity and group designation.

Data were entered into an Excel spreadsheet at WPMC. At the conclusion of the study, data were de-identified and sent to the statistician for analysis. Descriptive and inferential statistical analyses were performed with SPSS Statistics Version 25.0 (IBM, Armonk, NY) software. The chi-square test and independent samples t-test were used for group comparisons at an alpha level of 5%.

Based on the assumption that the treatment group would have twice the percent as the control group achieving the primary outcome of 150 min of aerobic exercise per week at the conclusion of the study, a sample size of 25 in each group was estimated for statistical significance at alpha equal to 0.05 and with power of 80% (beta = 0.20).

## Results

**Fig. 1:**
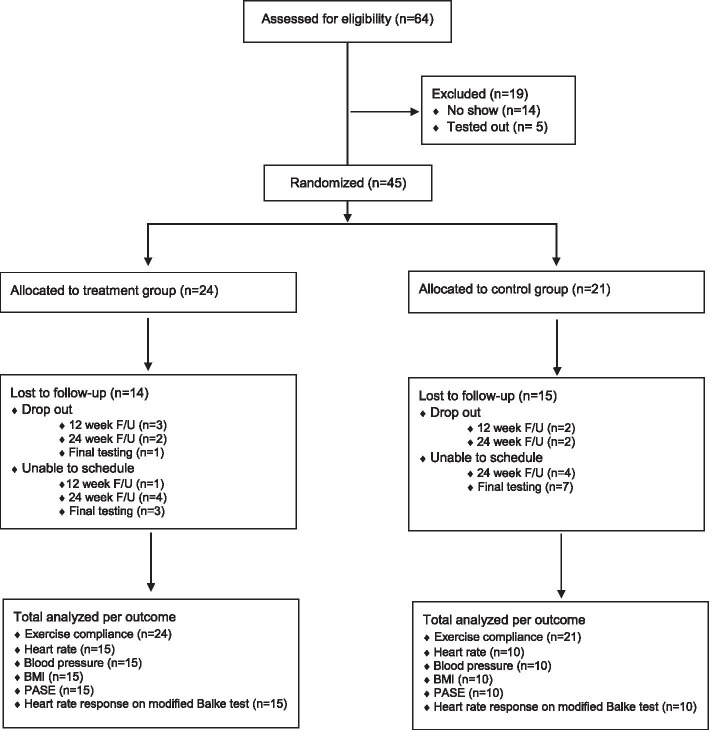
Flow diagram of participation

Sixty-four volunteers were recruited from Internal Medicine Clinic A at WPMC in October 2019. All volunteers were screened with a modified Balke treadmill test. With 14 volunteers not appearing for the treadmill testing and 5 volunteers unable to safely ambulate at 2.0 mph, 45 participants were eligible and agreed to participate. These participants were subsequently randomized to the exercise group (*n* = 24) and control group (*n* = 21). Figure 1 is a flow diagram of participation.

Table 2 shows that the baseline demographic and clinical characteristics were similar between the two groups. Follow-up at 12 weeks was completed by 39 of 45 participants (87%), at 24 weeks by 27 of 45 (60%), and at 34 weeks by 25 of 45 participants (55%).

**Table 2:** Demographic and clinical characteristics for the treatment and control groups

Demographic characteristics^a b^	Treatment ***n = 24***	Control ***n = 21***	***P***‡
Age, yr – mean ± sd	72.5 ± 7.2	73.3 ± 8.2	0.35
Male, *n*(%)	17 (70.8)	10 (47.6)	0.11
Caucasian, *n(%)*	21 (87.5)	19 (90.5)	1.00
**Clinical characteristics**^b^			
Weight, lb. – mean ± sd	208 ± 45.4	192.3 ± 45.6	0.26
BMI – mean ± sd	31.4 ± 5.5	31.0 ± 6.8	0.84
Resting heart rate, bpm – mean ± sd	72.8 ± 11.8	72.5 ± 10.1	0.92
Systolic blood pressure, mmHg – mean ± sd	135.8 ± 15.8	133.0 ± 18.8	0.59
Diastolic blood pressure, mmHg – mean ± sd	79.1 ± 8.5	76.0 ± 8.3	0.20
Physical Activity Score for the Elderly – mean ± sd	123.1 ± 46.6	132.4 ± 47.1	0.51
Obesity, *n(%)*	16 (66.7)	11 (52.4)	0.33
Diabetes mellitus, *n(%)*	12 (50.0)	6 (28.6)	0.14
Hypertension, *n(%)*	20 (83.3)	16 (76.2)	0.82
Dyslipidemia, *n(%)*	16 (66.7)	15 (71.4)	0.73
Obstructive sleep apnea, *n(%)*	6 (25.0)	7 (33.3)	0.54
Atrial fibrillation/flutter, *n(%)*	7 (29.2)	1 (4.8)	0.08
Congestive heart failure, *n(%)*	1 (4.2)	1 (4.8)	1.00
Coronary artery disease, *n(%)*	5 (20.8)	3 (14.3)	0.86
Peripheral vascular disease, *n(%)*	1 (4.2)	0	1.00
Stroke, *n(%)*	1 (4.2)	1 (4.8)	1.00
Chronic kidney disease, *n(%)*	4 (16.7)	5 (0.238)	0.82
Asthma, *n(%)*	1 (4.2)	1 (4.8)	1.00
Chronic obstructive pulmonary disease, *n(%)*	1 (4.2)	2 (9.5)	0.91
Anemia, *n(%)*	1 (4.2)	0	1.00
Malignancy, *n(%)*	2 (8.3)	1 (4.8)	1.00
Hypothyroidism, *n(%)*	0	3 (14.3)	0.19
Hyperthyroidism, *n(%)*	0	1 (4.8)	0.95
Kleinfelter syndrome, *n(%)*	1 (4.2)	0	1.00
Osteoporosis, *n(%)*	2 (8.3)	2 (9.5)	1.00
Osteoarthritis, *n(%)*	7 (29.2)	7 (33.3)	0.76
Rheumatoid arthritis, *n(%)*	0	1 (4.8)	0.95
Fibromyalgia, *n(%)*	0	1 (4.8)	0.95
Degenerative disc disease, *n(%)*	4 (16.7)	5 (23.8)	0.82
Spinal stenosis, *n(%)*	1 (4.2)	2 (9.5)	0.91
Depression, *n(%)*	1 (4.2)	3 (14.3)	0.51

*sd* Standard deviation

^a^All study participants lived either independently or with a spouse. ^b^Continuous variables are reported as mean ± standard deviation; categorical variables are reported as count (percentage). **‡**Independent samples t test for continuous variables; chi-squared test for categorical variables

The treatment group (*n* = 24) had better exercise compliance with 41.7% of participants reaching the goal of 150 min of weekly aerobic exercise at 34 weeks compared to 0% in the control group (*n* = 21) (*p =* 0.003) (Table 3).

**Table 3:** Comparison of treatment group and control group on outcomes

Outcome	Treatment Group	Control Group	P*
150 min of weekly aerobic exercise – n (%)^1^	10 (41.7)	0 (0.0)	0.003
Physical Activity Scale for the Elderly (0 to 400)			
Change: baseline to 34 weeks^2^	increase 66	decrease 20	< 0.001
Balke heart rate: change from Stage 1 to 6 - bpm			
Change: baseline to 34 weeks^2^	decrease 2.4	increase 5.3	0.038
Vitals check: heart rate			
Change: baseline to 34 weeks^2^	increase 6.9	increase 0.8	0.06
Vitals check: systolic blood pressure			
Change: baseline to 34 weeks^2^	decrease 5.9	decrease 0.3	0.49
Vitals check: diastolic blood pressure			
Change: baseline to 34 weeks^2^	decrease 0.4	increase 1.5	0.60
Vitals check: body mass index			
Change: baseline to 34 weeks^2^	decrease 0.16	increase 0.11	0.65

*Chi-squared test for 150 min of weekly aerobic exercise; independent samples t test for continuous variables

Sample size:

^1^Treatment = 24; Control = 21

^2^Treatment = 15; Control = 10

At 34 weeks, the treatment group (*n* = 15) had a mean increase in Physical Activity Score for the Elderly (PASE) of 66 points from baseline, whereas the control group (*n* = 10) had a mean decrease of 20 points (*p* < 0.001). For modified Balke testing, the treatment group had a mean decrease of 2.4 beats/min, whereas the control group had a mean increase in 5.3 beats/min (*p* = 0.038) (Table 3). No differences were observed between groups for mean change in heart rate (*p* = 0.06), systolic blood pressure (*p* = 0.49), diastolic blood pressure (*p* = 0.60), or body mass index (*p* = 0.65) (Table 3).

## Discussion

This 34-week, randomized cohort study with assessor blinding suggested that an unsupervised graded aerobic exercise protocol with biweekly monitoring is more effective than usual care for increasing exercise compliance among elderly individuals with chronic disease. Forty of our 45 participants had more than one comorbidity. Despite gym closures and limited access to exercise equipment during the COVID-19 pandemic, the treatment group had an impressive level of compliance with nearly 42% of participants achieving the goal of 150 min of weekly aerobic exercise. This finding is consistent with past studies demonstrating that patient participation in exercise was increased when physicians prescribed specific recommendations [4, 5]. Further, this result was achieved in a population with arguably the most physical and emotional obstacles to exercise and physiologic adaptation. Unhappily, the study began just prior to the arrival of COVID-19 in southwest Ohio, and the pandemic may well have lessened the effectiveness of the program. Nevertheless, our principal finding reinforces the importance of physicians assuming a more active role in promoting physical activity for their patients.

Participants in the treatment group benefited with respect to PASE and heart rate response on the modified Balke treadmill test. The PASE improvement reflected an increase in physical activities such as recreation, housework, home repairs, yard care, outdoor gardening, and volunteer work. The PASE improvement suggests that the protocol not only motivated more exercise, but also created an expectation for exercise to become a necessary part of one’s daily routine. In a 2019 study, a higher PASE was related to increased strength and muscle mass in elderly adults and was protective against frailty and sarcopenia [9]. Perhaps, most importantly, the improvement in the final PASE, taken in the midst of the COVID-19 pandemic, speaks to the consistency that our protocol fostered. Not only did participants in the treatment group improve their activity levels, but they were able to achieve peak conditioning when the barriers to exercise were the greatest. Similar to the increased PASE in the treatment group, the improvement in heart rate response on the modified Balke test demonstrated enhanced cardiorespiratory fitness, which has been found to reduces rates of all-cause and disease-specific mortality in multiple studies over the past 30 years [10]. Despite the exercise protocol being limited to low-intensity activity, participants were able to benefit from an increase in aerobic conditioning.

While the Balke change in heart rate from baseline to week 34 favored the treatment group over the control group by nearly 8 bpm (− 2.4 bpm vs. + 5.3 bpm), heart rate change from baseline to week 34 with the vitals check favored the control group over the treatment group by over 6 bpm (+ 0.8 bpm vs. + 6.9 bpm). This conflict in results was likely due to a deviation in protocol caused by lack of resources and time restrictions. Both treatment and control group participants were not allowed to wait the usual 5-min period to relax prior to measurement of their vitals during final testing. This deviation from the protocol may have led to artificially elevated heart rates that were not true representations of the participants’ cardiorespiratory fitness. We are obligated to report all findings, but believe the Balke measurements of heart rate were the more trustworthy.

Blood pressure change favored the treatment group over the control group (SBP: − 5.9 vs. -0.3 and DBP − 0.4 vs. + 1.5), but our study’s small sample size precluded these differences of 5.6 mmHg for SBP and 1.9 mmHg for DBP from being statistically significant. Also, COVID-19 restrictions resulted in different BP measuring devices being used at baseline, quarterly, and final measurements. Finally, BMI change also favored the treatment group (− 0.16 vs. + 0.11). This small 0.27 BMI benefit for the treatment group demonstrates the relative immutability of weight reduction in elderly adults when only 150 minutes of weekly aerobic exercise at a low intensity is prescribed.

To our knowledge, this study was the first to specifically evaluate the effect of a graded exercise protocol and biweekly monitoring on exercise compliance in elderly individuals with multiple comorbidities. Though numerous studies have evaluated the effectiveness of many different exercise protocols in various populations, patient compliance remains the principal barrier to exercise in the primary care setting. We developed a patient-centered approach to exercise that addresses many of the reported obstacles to exercise counseling and provides a time-efficient method that can be applied readily to outpatient practice. The notable improvements in patient adherence occurred with only a minimal time investment from physicians. We used only a single exercise handout in combination with a short biweekly phone call, both of which were simple and effective. Importantly, the simplicity of our approach lends itself to implementation by other members of the medical team to include nurses, medical assistants, or other support staff, which may be the best option for integration into a busy primary care practice. In addition, our program achieved favorable results in a difficult population of patients, many of whom had severe lack of cardiopulmonary reserve. Consequently, a similar approach may be even more effective in healthier populations with fewer barriers to exercise and more ability to adapt physiologically.

Though practical constraints limited our study to 34 weeks, it is feasible to use an exercise protocol with a similar rate of change over a much longer period. Once patient adherence is established, a similar model can be applied to greater exercise duration or used to maintain a patient’s current fitness level and preserve their functionality for years to come. Ideally, there would be regular follow-up appointments throughout the program to examine future goals. As mentioned previously, these follow-up appointments could be performed by other members of the medical team (nurse, medical assistant, etc.) instead of the physician, unless the exercise prescription requires significant modification. 

The most notable limitation of our study was COVID-19. The arrival of the pandemic led to an increase in the dropout rate for both groups, which limited the power of our study. Exercise compliance among those in the treatment group also decreased due to gym and mall closures as well as fear of leaving the house. Furthermore, the pandemic impacted our planned consistency for participant measurement. Prior to COVID-19, our plan was to perform all baseline, quarterly, and final measurements of heart rate, blood pressure, and BMI in our clinic with the same staff, protocols, and measuring devices. Similarly, we planned to perform the baseline and final modified Balke tests at the AFB’s gym on the same treadmill. However, when the base clinics and gym were closed, these measurements had to be performed at alternative locations and with unstandardized equipment having unknown calibrations. Consequently, measurement error was likely increased due to these unanticipated disruptions. Furthermore, limited hours of operation and equipment access during the pandemic prevented many participants from completing final testing. The chain of events caused by the COVID-19 pandemic also affected our pre-planned sample size designed to assure that clinically meaningful differences were also statistically significant. In addition, the study was conducted at a single military treatment facility. Thus, generalizability to other settings should be done with caution. Lastly, we acknowledge that our study design allowed for recall bias since it relied heavily on accurate reporting from the participants.

In conclusion, our graded aerobic exercise protocol with biweekly monitoring led to greater improvements in exercise compliance, PASE, and cardiorespiratory fitness when compared with usual care at 34 weeks in a population of elderly individuals with chronic disease. We believe that guided, independent exercise and surveillance can be an effective tool in primary care practice to help patients reach the recommended levels of exercise for both age and health status. However, given the small sample size in our study, future studies in elderly individuals with chronic disease are needed to confirm these findings. Future studies utilizing a team approach to patient exercise programming and surveillance with the possible addition of a fitness professional may lead to even greater changes in patient adherence and are strongly encouraged.   

## Data Availability

All data generated or analysed during this study in this published article and its supplementary information files.
